# Biomarker Testing, APOE4 Status and ARIA Risk Assessment In Alzheimer's Disease Patients: Insights from Australia and Hong Kong

**DOI:** 10.1002/alz70856_103681

**Published:** 2025-12-26

**Authors:** Georgia Giannakopoulou, Hannah Brown, Fleur Caponong, Joseph I Vindici, Sandra Teoh, Amber King, Pieter De Richter, Nazira Jainis, Hasifullah Ibrahim

**Affiliations:** ^1^ Ipsos, London, London, United Kingdom; ^2^ Ipsos, Sydney, NSW, Australia; ^3^ Ipsos, New York, NY, USA; ^4^ Ipsos, Kuala Lumpur, Kuala Lumpur, Malaysia; ^5^ Ipsos, Kuala Lumpur, Kuala Lumpur, United Kingdom

## Abstract

**Background:**

Biomarker testing and the risk assessment of amyloid‐related imaging abnormalities (ARIA) are crucial components in the patient selection and monitoring of disease‐modifying therapies (DMTs) for Alzheimer's disease (AD). This study evaluated the adoption of these practices in AD patients in Australia and Hong Kong (HK).

**Methods:**

A multi‐centre online survey of treating neurologists and geriatricians was conducted in Australia (July‐September 2024) and HK (November‐December 2024). De‐identified patient charts were recorded for patients with mild cognitive impairment, mild or moderate AD (suspected or confirmed) seen in the last 3 months. 29 HCPs in Australia and 15 in HK recorded biomarker use, APOE4 status and perceived ARIA risk of 64 and 45 patients, respectively. Recruited HCPs were screened for duration in specialty and caseload. HCP understanding of ARIA in the context of DMTs was collected in HK *(small base; qualitative findings)*.

**Results:**

The use of imaging and CSF biomarkers as diagnostic aids was limited among reported AD patients. In Australia, amyloid‐CSF (6.3%), amyloid‐PET (1.6%), tau‐CSF (3.1%), and tau‐PET (0%) tests were rarely conducted. In HK, amyloid‐PET was used in 2.2% of patients, with other biomarker PET or CSF tests not performed in any reported cases.

Aβ42/40 ratio and *p*‐tau biomarkers were also underutilized. In Australia, these tests were deemed not applicable for 73.4% and 75% of patients, respectively, with the testing status unknown for a further 17.2%. In HK, they were not applicable for 64.4% of reported patients and unknown for 13.3% (Aβ42/40 ratio) and 15.6% (*p*‐tau).

APOE4 status was unknown or not tested for all reported patients in Australia and 64.4% in HK. ARIA risk was unknown for 56.3% of patients in Australia, while in HK, 82.2% were cited as “no risk” and 4.4% as unknown. Yet, 73.3% of HK HCPs indicated a basic or limited understanding of ARIA.

**Conclusion:**

In this study cohort, biomarker testing, APOE genotyping and ARIA risk assessment were limited among AD patients in Australia and HK, potentially hindering a robust identification of DMT candidates. Future research should investigate the implementation of these practices in larger samples to help advise where further education is needed.

